# Role of NADPH Oxidase 4 in Corneal Endothelial Cells Is Mediated by Endoplasmic Reticulum Stress and Autophagy

**DOI:** 10.3390/antiox12061228

**Published:** 2023-06-07

**Authors:** Dae Joong Ma, Jin Sun Hwang, Kyung Bo Noh, Sun-Hee Oh, Kyoung Wook Kim, Young Joo Shin

**Affiliations:** 1 Department of Ophthalmology, Hallym University Medical Center, College of Medicine, Hallym University, Seoul 07442, Republic of Korea; 2 Hallym BioEyeTech Research Center, College of Medicine, Hallym University, Seoul 07442, Republic of Korea

**Keywords:** NOX4, corneal-endothelial cells, autophagy, reactive oxygen species, senescence, in vivo transfection

## Abstract

Human corneal-endothelial cells (hCEnCs) are located on the inner layer of the cornea. Injury to CEnCs leads to permanent corneal edema, requiring corneal transplantation. NADPH oxidase 4 (NOX4) has been reported to be implicated in the pathogenesis of CEnCs diseases. Thus, we investigated the role of NOX4 in CEnCs in this study. In an animal study, siRNA for NOX4 (siNOX4) or plasmid for NOX4 (pNOX4) was introduced into the corneal endothelium of rats by electroporation, using a square-wave electroporator (ECM830, Havard apparatus) to decrease or increase the expression of NOX4, respectively, and the rat corneas were cryoinjured through contact with a metal rod of 3 mm diameter frozen in liquid nitrogen for 10 min. The immunofluorescence staining of NOX4 and 8-OHdG showed that the levels of NOX4 and 8-OHdG were decreased in the siNOX4 group compared to the siControl, and increased in the pNOX4 group compared to the pControl at one week after treatment. Without cryoinjury, corneal opacity was more severe, and the density of CEnCs was lower, in pNOX4-treated rats compared to pControl. After cryoinjury, the corneas were more transparent, and the CEnC density was higher, in siNOX4-treated rats. The hCEnCs were cultured and transfected with siNOX4 and pNOX4. The silencing of NOX4 in hCEnCs resulted in a normal cell shape, higher viability, and higher proliferation rate than those transfected with the siControl, while NOX4 overexpression had the opposite effect. NOX4 overexpression increased the number of senescent cells and intracellular oxidative stress levels. NOX4 overexpression increased ATF4 and ATF6 levels, and nuclear translocation of XBP-1, which is the endoplasmic reticulum (ER) stress marker, while the silencing of NOX4 had the opposite effect. Additionally, the mitochondrial membrane potential was hyperpolarized by the silencing of NOX4, and depolarized by NOX4 overexpression. The LC3II levels, a marker of autophagy, were decreased by the silencing of NOX4, and increased by NOX4 overexpression. In conclusion, NOX4 plays a pivotal role in the wound-healing and senescence of hCEnCs, by modulating oxidative stress, ER stress, and autophagy. The regulation of NOX4 may be a potential therapeutic strategy for regulating the homeostasis of CEnCs, and treating corneal-endothelial diseases.

## 1. Introduction

Oxidative stress plays a crucial role in the pathogenesis of corneal-endothelial diseases [1]. Human corneal-endothelial cells (hCEnCs) require a large amount of energy to keep the cornea dehydrated and transparent by pumping out water from the cornea [2]. Thus, they generate reactive oxygen species (ROS) as a byproduct of energy production [3]. ROS are generated by biochemical reactions that occur during the processes of respiration in organelles such as mitochondria and peroxisomes [4]. Excessive ROS levels cause oxidative cellular damage to the DNA, proteins, and lipids. Additionally, excess ROS impair physiological functions, and are involved in the pathogenesis of various diseases including cancers, neurodegenerative disorders, cardiovascular diseases, and aging [5]. ROS are removed via an antioxidative system including enzymatic and non-enzymatic antioxidants [6]. The enzymatic antioxidants are glutathione reductase, superoxide dismutase, catalase, and ascorbate peroxidase [6]. The non-enzymatic antioxidants include glutathione, flavonoids, carotenoids, tocopherol, and phenolic compounds [6]. Oxidative stress is linked to the ER stress and autophagy [7] that have been reported to be involved in the pathogenesis of corneal-endothelial diseases [8,9,10,11]. In the ER, the proteins newly synthesized by the ribosomes are folded into three-dimensional structures from a linear amino-acid sequence [12]. The failure of protein-folding results in the accumulation of unfolded or misfolded protein in the ER, which causes protein apoptosis and pathophysiological changes [13]. Autophagy is an intracellular process for the removal of abnormal protein and damaged organelles by lysosomes [14]. Mitochondrial autophagy in Fuchs’ endothelial corneal dystrophy (FECD) leads to a decrease in mitochondrial mass and dysfunction. The inhibition of autophagy flux can elevate the mitochondrial mass in CEnCs [3,15]. Oxidative stress can be linked to ER stress and autophagy in several signaling pathways [16].

NADPH oxidases (NOXs) are a family of enzymes that are located in the plasma membrane of cells. They are multiple-subunit complexes that work to catalyze the transfer of electrons from NADPH or NAPH to oxygen, to generate O_2_^−^ and H_2_O_2_ [17,18]. NADPH oxidase 4 (NOX4) generates ROS [19], which is metabolized by peroxiredoxin 4 (PRDX4) in the endoplasmic reticulum (ER) lumen [20]. The dysregulation of NOX4 activity leads to an elevated ROS production that can contribute to cell proliferation, cell death, or epithelial-to-mesenchymal transition [21]. NOX4 promotes proliferation in vascular endothelial cells [22], but inhibits cell proliferation, and induces cell death in hepatocytes and other cells [23,24]. Additionally, a recent study reported increased NOX4 expression in corneal-endothelial diseases including FECD, compared to a normal cornea [25]. NOX4-produced ROS can induce the senescence and subsequent activation of the nuclear factor kappa-light-chain-enhancer of activated B cells (NF-κB), and production of cytokines and chemokines [17,26]. However, the specific role of NOX4 and NOX4-produced ROS in the corneal endothelium has not been extensively reported. This study aimed to investigate the specific role of NOX4 on hCEnCs.

## 2. Materials and Methods

### 2.1. Animal Study

This study was approved by the Institutional Animal Care and Use Committee of Hallym University Medical Center. All procedures were performed according to the Association for Research in Vision and Ophthalmology Statement for the Use of Animals in Ophthalmic and Vision Research. Six-week-old female Sprague-Dawley (SD) rats (Raonbio, Yongin, Republic of Korea) were used for this procedure. Six SD rats were included in each group.

### 2.2. In Vivo Transfection and Evaluation

siRNA (1 nmol) and DNA (0.1 nmol) were introduced into the anterior chamber of SD rats, followed by the placement of 7 mm Tweezertrodes (BTX Harvard Apparatus, Holliston, MA, USA) on both corneas. The positive electrode was placed on the eye that received the gene injection. The ECM830 electroporation system (BTX Harvard Apparatus) was used. The parameters were 140 V, 100 ms length, 950 ms interval, 5 pulses, and 100 V/cm^2^. The corneal endothelium was cryoinjured for 10 s with a metal rod of 3 mm diameter frozen in liquid nitrogen for 10 min, followed by irrigation with isotonic sodium chloride solution. The corneal opacity was graded using photographs obtained on days 2, 4, 7, 9, 11, and 14. Corneal opacity was evaluated as previously described [27]. Alizarin Red S staining was conducted with 0.2% Alizarin Red S (pH 4.2) for 90 s. 2.5% glutaraldehyde was used to fix the corneas. The corneas were excised, and placed on the slides. The corneal endothelium was observed under the microscope (DMi8; Leica, Wetzlar, Germay). The transfection efficiency was calculated after the in vivo transfection of green fluorescent protein (GFP)-encoded plasmid (sc-108083) and GFP-conjugated siRNA (Bioneer, Seoul, Republic of Korea).

### 2.3. Cell Culture and Transfection

This study was reviewed and approved by the institutional review board/ethics committee of the Hallym University Medical Center (NON2022-007) because the corneas from human cadaveric donors were used. The hCEnCs were cultured as previously reported [28]. Briefly, human corneas were obtained from Eversight (Ann Arbor, MI, USA). The hCEnCs and Descemet’s membrane complexes was harvested and incubated in a growth medium at 37 °C in an incubator overnight. The next day, the hCEnCs were trypsinized and seeded in a 6-well culture plate. The hCEnCs were transfected with the siRNA against NOX4 (siNOX4, 5′-CUGUUG UGGACCCAAUUCA-3′, and 5′-UGAAUUGGGUCCACAACAG-3′; Bioneer Corp., Daejeon, Republic of Korea), or the control (Bioneer Corp.; siControl), using Lipofectamine™ RNAiMAX reagent (Invitrogen, Waltham, MA, USA). The pcDNA3.1-human NOX4 was obtained from Addgene (Karl-Heinz Krause, #69352; http://n2t.net/addgene:69352 (accessed on 1 October 2020); RRID: Addgene_69352; Watertown, MA, USA). The plasmids for NOX4 (pNOX4) were transfected into the cultured hCEnCs using the Lipofectamine™ 3000 reagent (Invitrogen). After incubation for 48 h, the cells were harvested. The NOX4 expression was confirmed by RT-PCR 48 h after transfection. The primers used were GACTTTACAGGTATATCCGGAGCAA for NOX4 forward, and TGCAGATACACTGGACAATGTAGA for NOX4 reverse (gene accession number: NM_001143836).

### 2.4. Cell Viability and Proliferation Assay

The cells (1 × 10^4^) were placed in 96-well plates. Cell viability was evaluated using a Cell Counting kit-8 (CCK-8; Dojindo, Kumamoto, Japan). The cells were treated with a CCK-8 reagent for 1 to 2 h. The absorbance at 450 nm was measured using a microplate reader (Synergy HTX, BioTek, Winooski, VT, USA), to determine cell viability.

The cell proliferation rate was evaluated using a commercial bromodeoxyuridine (BrdU) proliferation assay kit (Roche Diagnostics, Mannheim, Germany). The cells (5 × 10^3^ cells/well) were cultured in 96-well plates and treated with BrdU labeling. After treating the plate in FixoDent reagent for 30 min at room temperature (RT), the cells were treated with the anti-BrdU-POD reagent for approximately 90 min at RT. The substrate reagent was then added, and the wells were treated for 20 min at RT. Then, 1 M H_2_SO_4_ was added to stop the reaction. The optical density at 450 nm measured using a microplate spectrophotometer (Synergy HTX, BioTek) was used to evaluate the proliferation rates after the subtraction of the corresponding blanks.

The cell shape was evaluated after transfection. The elongation factor was calculated, to determine the extent of cell-shape changes. The elongation factor is a parameter used to quantify the degree of elongation or deformation in cell morphology. After images of the cells were acquired, Axiovert 4.7 was used to measure the cell length and width. The elongation factor is calculated using the formula: Elongation Factor = Length/Width.

### 2.5. Cell Cycle Analysis

Muse Cell Analyzer (Merck Millipore, Burlington, MA, USA) with propidium iodide (PI) staining was used to analyze the cell cycle. Briefly, cells were trypsinized and washed with ice-cold phosphate-buffered saline (PBS). Then, 70% ethanol was used for overnight fixing at −20 °C. The fixed cells were collected, and rinsed with PBS. A solution containing PI (50 μg/mL) and RNase A (100 μg/mL) was added, and incubated for 30 min at RT in the dark.

### 2.6. Immunofluorescent Staining

The hCEnCs were cultured on cell-culture slides (SPL Life Sciences, Seoul, Republic of Korea). The cells and corneas were fixed for 20 min in a 4% paraformaldehyde solution, permeabilized for 10 min with 0.5% Triton X-100, and blocked for 1 h with 1% bovine serum albumin (BSA) at RT. The samples were incubated overnight with mouse anti-NOX4 antibody (MABC616, Merck Millipore, Burlington, MA, USA), mouse anti-8-OHdG antibody (ab62623, Abcam, Cambridge, UK), rabbit anti-human NF-κB antibody (sc-372; Santa Cruz Biotechnology, Santa Cruz, CA, USA) or rabbit anti-X-box binding protein 1 (XBP-1) antibody (sc-7160, Santa Cruz Biotechnology) at 4 °C. The cells were treated with fluorescein isothiocyanate-conjugated goat anti-rabbit IgG antibody (1:100) for 2 h at RT in the dark, followed by counterstaining with Hoechst 33,342 dye (1:2000; Molecular Probes, Eugene, OR, USA). The slides were viewed under a fluorescence microscope (DMi8; Leica).

### 2.7. Western Blot

The radioimmunoprecipitation assay buffer, (Biosesang, Seoul, Republic of Korea) including protease (Roche, Basel, Switzerland) and phosphatase (PhosSTOP; Roche) inhibitor cocktails, was employed to extract the total cellular proteins. Western blotting was performed using standard protocols. Nonspecific binding was blocked with 5% skim milk for 1 h. The primary antibodies were mouse anti-activating transcription factor 4 (ATF4; sc-390063, 1:500 dilution; Santa Cruz), anti-activating transcription factor 6 (ATF6; PA5-20215, 1:500 dilution; Thermo Fisher, Waltham, MA, USA), rabbit anti-XBP-1 antibody (sc-7160, Santa Cruz), mouse anti-LC3 (M186-3, 1:1000 dilution; MBL International Corporation, Woburn, MA, USA), or rabbit anti-GAPDH (LF-PA0212, 1:5000 dilution; AbFrontier Co., Ltd., Seoul, Republic of Korea). Horseradish peroxidase (HRP)-conjugated secondary antibody and a WEST-Queen™ Western Blot Detection Kit (iNtRON Biotechnology, Seongnam, Republic of Korea) were employed to detect the immunoreactive bands.

### 2.8. Real-Time Reverse Transcription-Polymerase Chain Reaction (qRT-PCR)

The RNA was extracted using the ReliaPrep™ RNA Miniprep Systems (Promega, Madison, WI, USA) [29]. First, first-strand cDNA was synthesized from 200 ng of oligonucleotide primers, using a commercially available kit (GoScript Reverse Transcription System; Promega). The real-time quantification of transcripts was performed on a LightCycler^®^ 96 (Roche Life Science, Mannheim, Germany) using the AccuPower 2X GreenStar qPCR Master Mix (Bioneer). The quantitative real-time RT-PCR data are presented as fold-changes relative to the control. The relative quantification was performed using the comparative CT or ΔΔCT methods. The target gene was normalized to β-actin. The primers are described in Supplementary Table S1.

### 2.9. Senescence-β-Galactosidase Assay

Senescence-β-galactosidase (SA-β-gal) staining was performed using the SA-β-gal staining kit (BioVision, Waltham, MA, USA). The cells were fixed for 15 min at RT, followed by the incubation of samples overnight at 37 °C in a staining reagent containing 5-bromo-4-chloro-3-indoyl-β-galactopyranoside at pH 6.0. The blue color indicated the presence of SA-β-Gal activity. The cells were observed under a microscope (DMi8; Leica, Wetzlar, Germany).

### 2.10. Mitochondrial Oxidative Stress Evaluation

MitoSOX^TM^ Red (5 μM; Invitrogen) was used to measure the mitochondrial superoxide production. The fluorescence intensity was assessed using a Cytoflex analyzer (Beckman Coulter Life Sciences, Indianapolis, IN, USA) at an excitation wavelength of 510 nm, and emission wavelength of 590 nm.

### 2.11. MitoPotential Assay

The Muse™ MitoPotential assay (Merck Millipore) was used to assess mitochondrial membrane potential. A total of 2.5 × 10^5^ cells were harvested by centrifugation (2000× *g* rpm, 3 min). The cell pellets were stained using the Muse MitoPotential Kit (Merck Millipore) for 25 min at 37 °C. The data were analyzed using the Muse™ Cell Analyzer.

### 2.12. Cellular ER Imaging

For the ER organelle imaging, photostable ER-Tracker Red staining (Invitrogen) was used. Briefly, cells were seeded in 12-well plates and stained with a 1 μM ER-tracker probe for 1 h in the dark (37 °C, 5% CO_2_). After washing with PBS, the cells were viewed under a microscope (DMi8; Leica).

### 2.13. Statistical Analysis

The data are expressed as mean ± standard deviation (S.D.). An independent t-test was used to compare the two groups. GraphPad Prism 9.0 (GraphPad Software, La Jolla, CA, USA) was used.

## 3. Results

### 3.1. Gene Transfection of the Rats, and the Effect of NOX4 on the Regulation of Corneal-Endothelial Cell Death In Vivo

**Figure 1:**
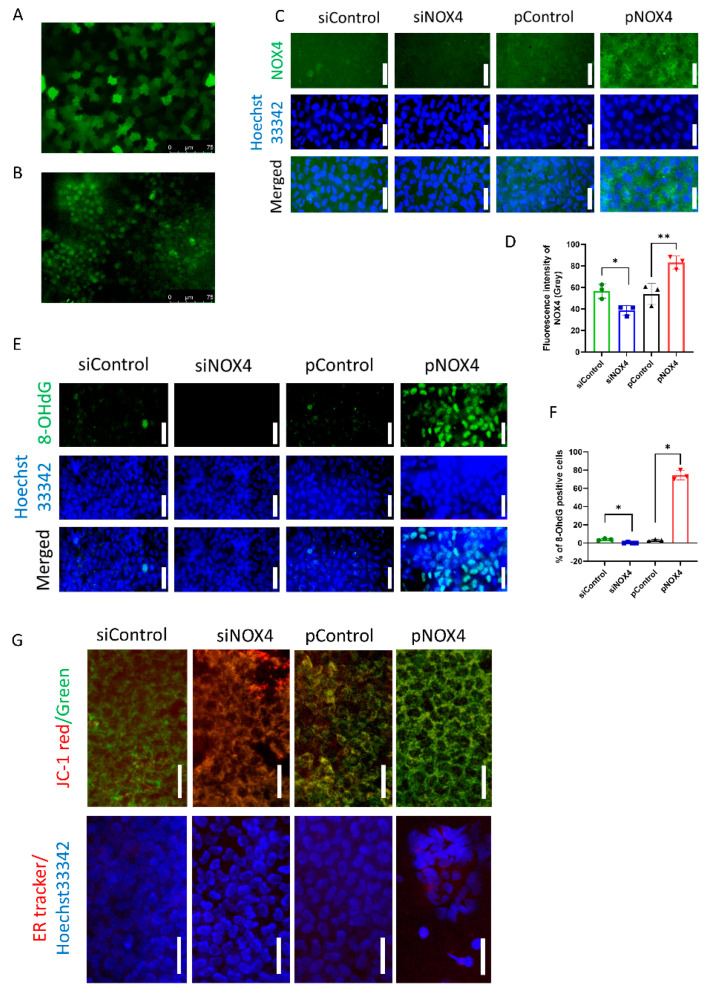
The in vivo transfection of genes. (**A**,**B**) The transfection efficiency using electroporation was evaluated with pGFP and fluorescein-conjugated siRNA. (**C**,**D**) The levels of NOX4 were evaluated using the immunofluorescence staining of NOX4 at one week after transfection. Scale bar = 150 µm. (**E**,**F**) The levels of 8-OHdG were evaluated using immunofluorescence staining of 8-OHdG at one week after transfection. Scale bar = 100 µm. (**G**) The in vivo staining of JC-1 and ER-Tracker red was performed to evaluate the mitochondrial membrane potential and ER stress. Scale bar = 100 µm * *p* < 0.05, ** *p* < 0.01.

To evaluate the transfection efficiency of in vivo gene delivery using electroporation, pGFP and fluorescein-conjugated siRNA were introduced into the corneal endothelium of rats. The transfection efficiency of the pGFP was 81.89 ± 7.83%, and that of the fluorescein-conjugated siRNA was 76.80 ± 17.25% (Figure 1A,B). The immunofluorescence staining of NOX4 and 8-OHdG was performed to evaluate NOX4 levels and oxidative stress levels. NOX4 was suppressed in the siNOX4 group compared to the siControl, and overexpressed in the pNOX4 compared to the pControl (Figure 1C,D). The number of 8-OHdG-stained cells was reduced in the siNOX4 group compared to the siControl, and elevated in the pNOX4 compared to the pControl (Figure 1E,F). JC-1 staining was conducted to assess the mitochondrial membrane potential of the rat corneal endothelium in vivo (Figure 1G). The mitochondrial membrane potential was increased in the siNOX4 group compared to the siControl, while it was decreased in the pNOX4 group compared to the pControl. ER-Tracker red staining was performed to evaluate the ER stress of the rat corneal endothelium in vivo (Figure 1G). The fluorescence intensity of ER-Tracker red was decreased in the siNOX4 group compared to the siControl, while it was increased in the pNOX4 group compared to the pControl.

**Figure 2:**
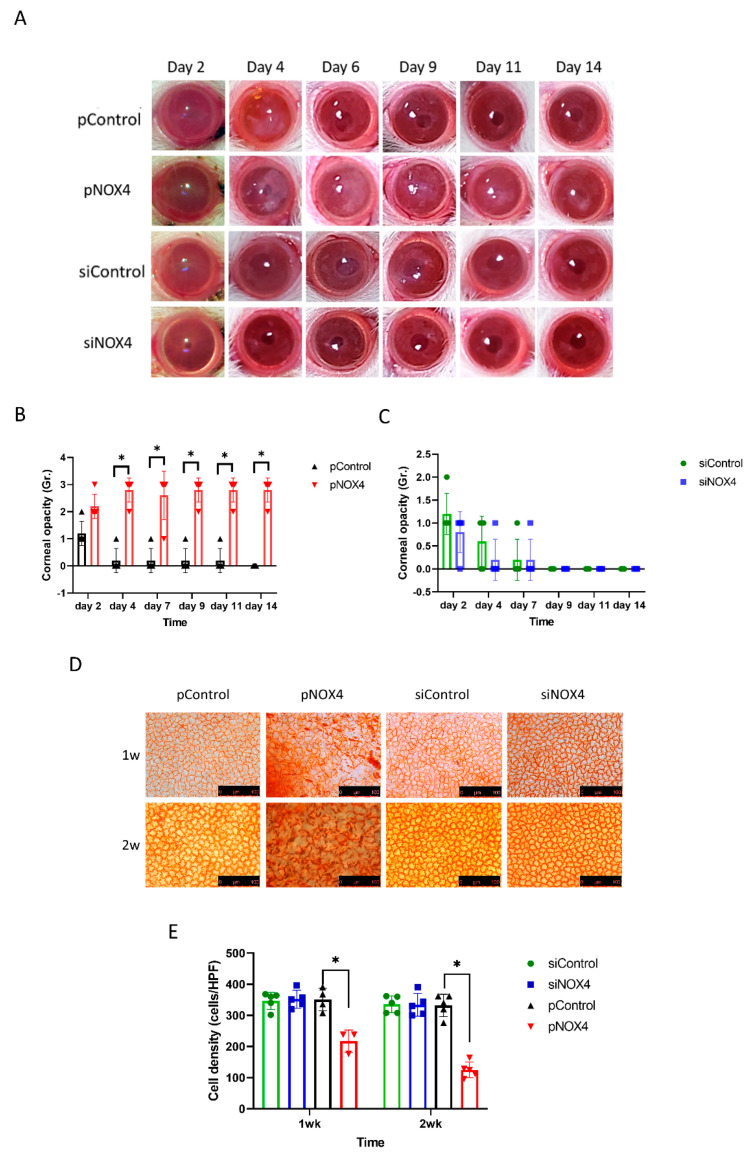
NOX4 regulates the cell death of corneal-endothelial cells in rat. (**A**–**C**) The corneas were evaluated after transfections of siNOX4 or pNOX4. The corneal opacity was graded. (**D**,**E**) Alizarin S red staining showed the corneal-endothelial cell density at one week and two weeks. * *p* < 0.05.

To evaluate the role of NOX4, the corneal endothelium of SD rats was transfected with either siNOX4 or pNOX4 (siRNA and a plasmid carrying NOX4, respectively) to inhibit or increase the expression of NOX4, respectively, in vivo, using electroporation without injury, followed by the evaluation of corneal opacity and histological changes. The corneal opacity was more severe in the pNOX4-treated rats compared to the pControl from day 4 to day 14 (Figure 2A,B), while the corneal opacity in the siNOX4 group was not different from the siControl group (Figure 2C). The corneas transfected with siNOX4 remained transparent. The cell density of the corneal endothelium, measured by Alizarin S red staining, was lower in the pNOX4-treated corneas compared to the pControl at one week and two weeks, while the cell density in the siNOX4 group was not different from the siControl group (Figure 2D,F).

**Figure 3:**
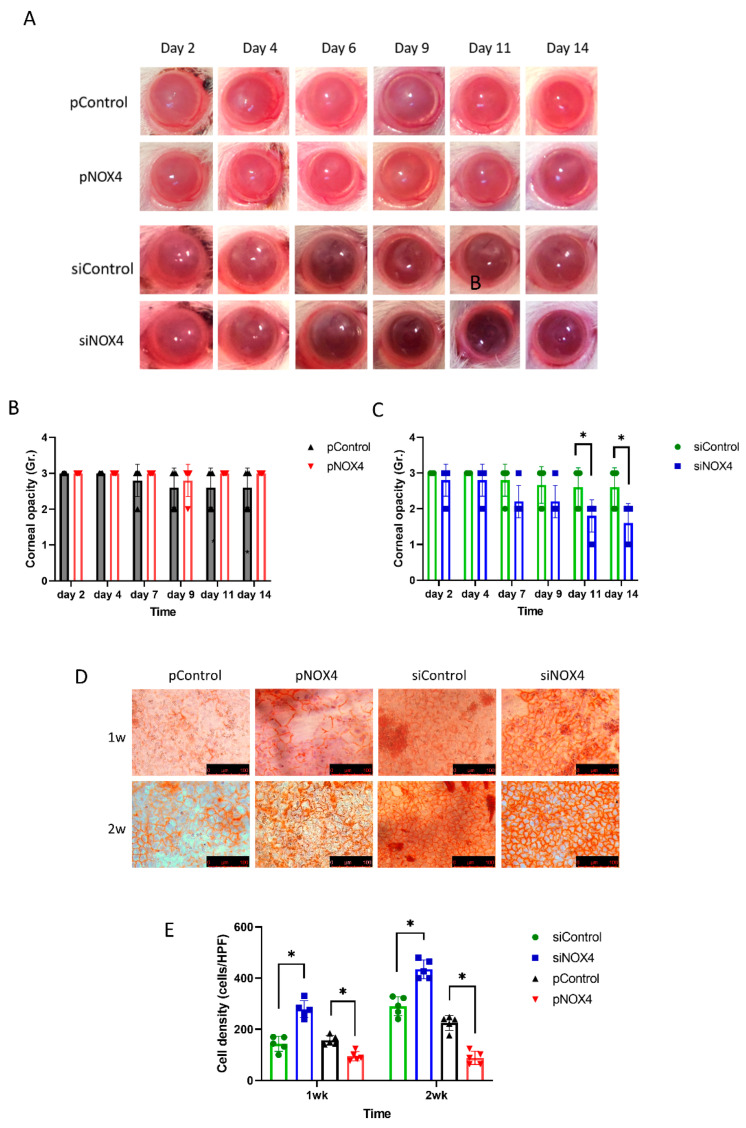
The effect of NOX4 on cryo-injured cornea. (**A**–**C**) Corneas were injured with cryoinjury after transfections of siNOX4 or pNOX4. Corneal opacity was evaluated. (**D**,**E**) Corneal-endothelial cells stained with Alizarin S red solution showed a recovery rate. * *p* < 0.05.

The corneal endothelium of SD rats was transfected and then injured by cryoinjury. The corneal opacity was reduced among the siNOX4-treated rats compared to the control groups at day 11 and day 14 (Figure 3A,C). The pNOX4 and pControl groups showed severe corneal opacity over the 14 days, and there was no difference between the pNOX4 and pControl groups (Figure 3B). The cell density of the rat corneal endothelium was higher in the siNOX4-treated corneas compared to the siControl, and lower in the pNOX4-treated corneas compared to the pControl (Figure 3D,F).

### 3.2. NOX4 Regulates the Cell Shape

**Figure 4:**
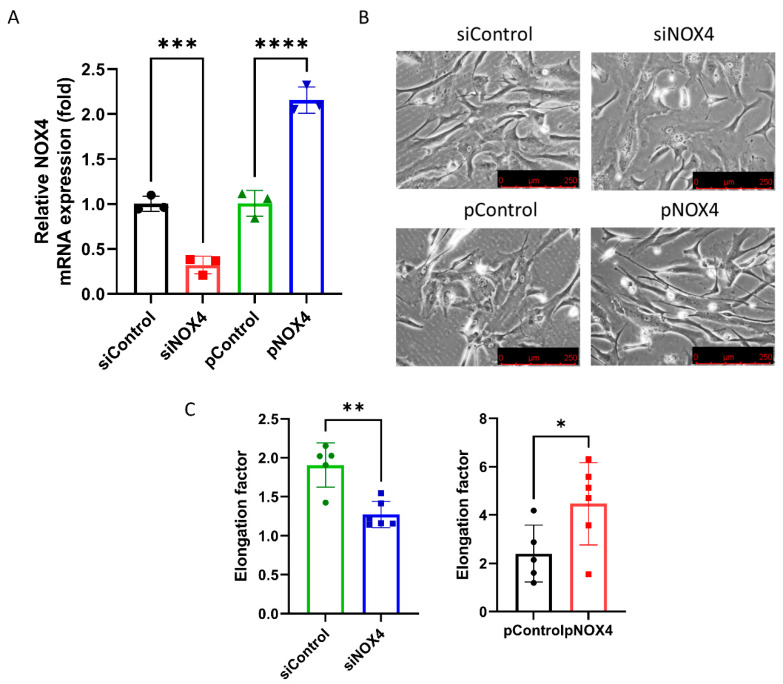
NOX4 regulates the cell shape and cell proliferation. (**A**) NOX4 mRNA expressions were evaluated after the transfection of siNOX4 or pNOX4 at 72 h after treatment. (**B**,**C**) The cell shape was observed using an inverted microscopy. The cell shape was evaluated using the elongation factor. Data presented as mean ± S.D. * *p* < 0.05, ** *p* < 0.01, *** *p* < 0.001 and **** *p* < 0.0001.

To assess the effect of siNOX4 and pNOX4, we measured the NOX4 mRNA levels in the treated cells. The NOX4 mRNA levels were reduced in the siNOX4-treated cells (68.1% reduction), and increased in the pNOX4-treated cells (115.0% increase; Figure 4A). To investigate the role of NOX4 on the cell morphology, we evaluated the cell shape using phase-contrast microscopy. The siNOX4-treated cells were round and short, whereas the pNOX4-treated cells were slender and fibroblast-like (Figure 4B). The cell shape was evaluated using the elongation factor. The cells were less elongated in the siNOX4 group compared to the siControl, and more elongated in the pNOX4 group compared to the pControl (Figure 4C).

### 3.3. NOX4 Regulates Senescence by Regulating ROS Levels

**Figure 5:**
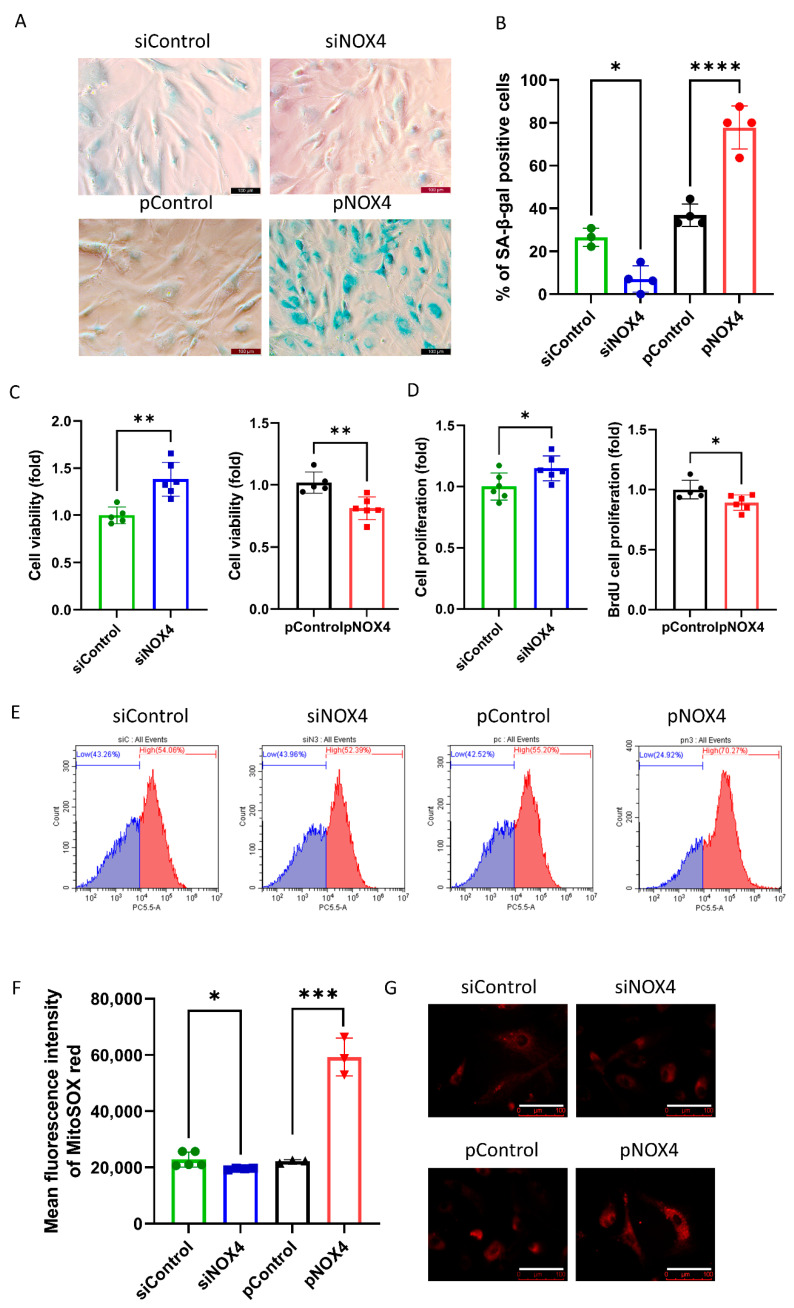
NOX4 regulates senescence. (**A**,**B**) A senescence-associated β-galactosidase assay was performed as a senescence marker at 72 h after treatment. (**C**) Cell viability was measured using the CCK-8 kit. (**D**) The cell proliferation rate was measured by a BrdU incorporation assay. (**E**–**G**) Mitochondrial oxidative stress levels were measured using a MitoSOX probe. Scale bar = 100 µm. Data presented as mean ± S.D. * *p* < 0.05, ** *p* < 0.01, *** *p* < 0.001 and **** *p* < 0.0001.

SA-β-gal staining was used to evaluate senescence at 72 h after treatments. The results revealed that senescence was reduced in the siNOX4-treated cells, but increased in the pNOX4-treated cells at 72 h after treatments (Figure 5A,B). To investigate the role of NOX4 on cell proliferation, we performed a cell-viability assay using a CCK-8 and BrdU incorporation assay at 72 h after treatment. Cell viability was increased in the siNOX4-treated cells (38.2%, *p* = 0.024), but decreased in the pNOX4-treated cells (20.8%, *p* = 0.026; Figure 5C) at 72 h after treatments. The BrdU proliferation rates were increased in the siNOX4-treated cells (14.91%, *p* = 0.036), but decreased in the pNOX4-treated cells (10.9%, *p* = 0.030; Figure 5C) at 72 h after treatments. To evaluate the oxidative stress levels, MitoSOX probe was used. The mean fluorescence intensities of MitoSOX, intracellular oxidative stress levels, were lower in the siNOX4-treated cells compared to the siControl, and higher in the pNOX4-treated cells compared to the pControl (Figure 5E,F). These results indicate that NOX4 regulates senescence by regulating ROS levels.

### 3.4. NOX4 Regulates the ATF4 and XBP-1 Pathways during ER Stress

**Figure 6:**
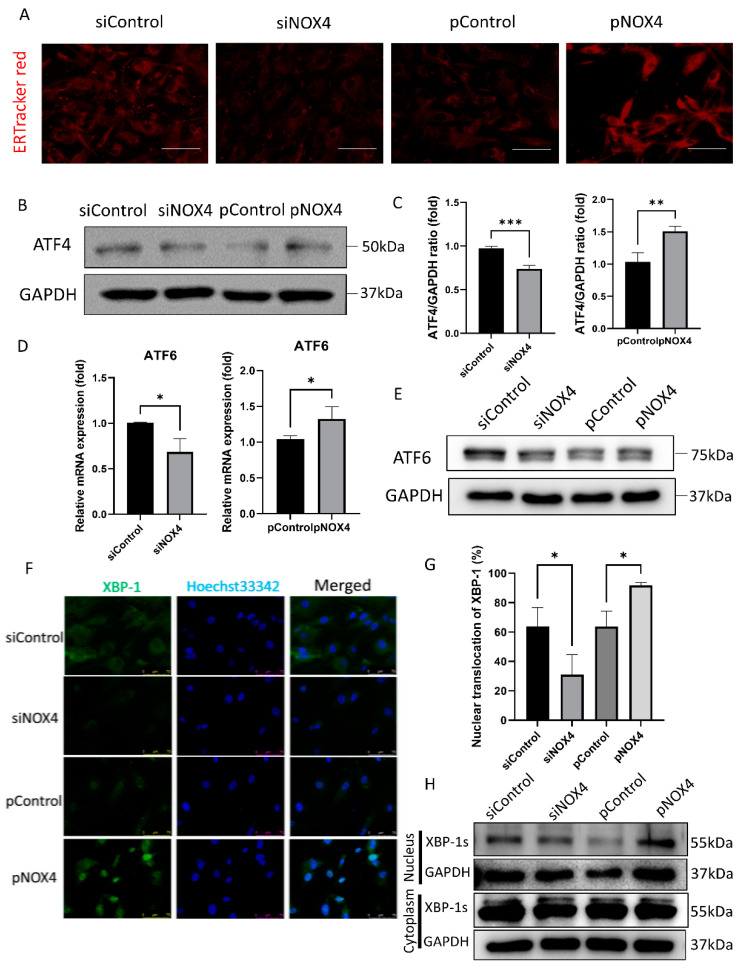
NOX4 regulates the ATF4 and XBP-1 branches of ER stress. (**A**) ER organelle visualization by ER-Tracker^TM^ red fluorescence imaging at 72 h after treatment. Scale bar = 100 µm. (**B**,**C**) ATF4 levels were evaluated by Western blotting, after transfections of siRNA for NOX4, or plasmids for NOX4. (**D**,**E**) RT-qPCR and Western blotting analyses for ATF6 were performed. (**F**,**G**) Immunofluorescence staining of XBP-1 was performed to evaluate the XBP-1 nuclear translocation. (**H**) Western blotting of XBP-1 was performed to evaluate the translocation of XBP-1. Data presented as mean ± S.D. * *p* < 0.05, ** *p* < 0.01, and *** *p* < 0.001.

To assess ER stress levels, we performed ER-Tracker Red staining, Western blotting of ATF4, and immunofluorescence staining of XBP-1. The ER-Tracker Red staining showed ER swelling and enlargement, which was increased in the pNOX4-treated cells, but reduced in the siNOX4-treated cells (Figure 6A). ATF4 and ATF6, which are activated during ER stress, were reduced in the siNOX4-treated cells, but increased in the pNOX4-treated cells (Figure 6B–E). The nuclear translocation of XBP-1 was decreased in the siNOX4-treated cells, and increased in the pNOX4-treated cells (Figure 6F,G), which was confirmed by Western blotting (Figure 6H). These results suggest that NOX4 regulates the ATF4 and XBP-1 pathways during ER stress.

### 3.5. NOX4 Regulates Autophagy through the Mitochondria

**Figure 7:**
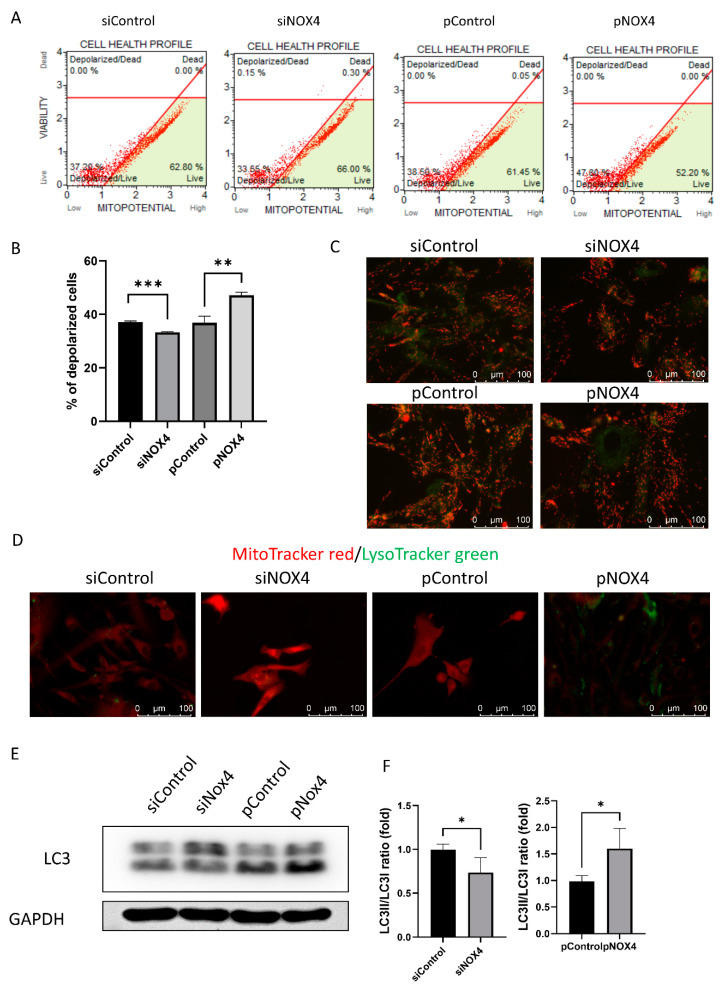
NOX4 regulates autophagy through the mitochondria. (**A**,**B**) The mitochondrial membrane potential was measured using MitoPotential kit at 72 h after treatment. (**C**) The JC-1 probe was used for obtaining images of the mitochondrial membrane potential. (**D**) Lysosome was visualized by LysoTracker green, and mitochondria was visualized by MitoTracker red. (**E**,**F**) Autophagy was evaluated by the Western blot of LC3. Data presented as mean ± S.D. * *p* < 0.05, ** *p* < 0.01 and *** *p* < 0.001.

To investigate the cell death and autophagy, we evaluated the mitochondrial membrane potential using JC-1, LysoTracker green staining, and Western blotting of LC3. The mitochondrial membrane potential was hyperpolarized in the siNOX4-treated cells, and depolarized in the pNOX4-treated cells (Figure 7A–C). Lysosomes stained with LysoTracker green were prominent in the pNOX4-treated cells (Figure 7D). Additionally, LC3II, a marker of autophagy, was increased in the pNOX4-treated cells (Figure 7E), indicating that NOX4 regulates autophagy.

## 4. Discussion

CEnCs are different to vascular endothelial cells. CD31, which is a marker for vascular endothelial cells, ref. [30], has not previously been employed for a study of the phenotype of CEnCs. CenCs are derived from mesenchymal cells from the neural crest [31]. They are sensitive to injury, and do not have the ability to regenerate [32]. It is therefore important to maintain the integrity of these cells, to ensure the clarity and health of the cornea. NOXs are the plasma membrane-bound enzymes that catalyze the transfer of electrons from NAPDH or NAPH to oxygen, to generate O_2_^−^ and H_2_O_2_ [17,18]. NOX4 functions as a mitochondrial energetic sensor, and is a major source of oxidative stress [18]. Mitochondria and oxidative stress play an important role in the function and survival of hCEnCs [33]. NOX4 overexpression results in excessive ROS production, thereby causing mitochondrial dysfunction and decreased barrier function [34,35], which plays an important role in the function and survival of CEnCs. In this study, we investigated the specific role of NOX4 on CEnCs, by inhibiting or increasing its expression, using siNOX4 and pNOX4, respectively.

In this study, to investigate the role of NOX4 on CEnCs in vivo, we introduced pNOX4 and siNOX4 into the rat corneal endothelium using electroporation, a method that has been suggested to effectively introduce genes into tissues without significant injuries [36,37]. However, corneal opacity was observed in all groups at day 2, which may be due to the side effects of electroporation, including the changes in temperature, pH, and electric field strength, and the formation of toxic substances [38]. This side effect disappeared rapidly. The in vivo transfection of siNOX4 and pNOX4 into the corneal endothelium of rats showed that NOX4 overexpression causes corneal opacity, and reduces cell density, despite no cryoinjury. Generally, the effects of siRNA-mediated gene silencing can last from a few days to several weeks. The persistence of the downregulation effect depends on the stability of the siRNA within the cell, and the turnover rate of the protein. CEnCs maintain corneal transparency by dehydrating the corneal stroma [39], which depends on mitochondrial energy production, and is hindered by excessive ROS generation [33,40]. CEnCs were migrated to cover the injured area, and restore the cells morphology, by 48 h after the injury [41]. It takes several days for CEnCs to restore their function, and for the cornea to regain clarity [32]. An impaired CEnC function causes the cornea to become opaque [39], and reduced cell density results in insurmountable decompensation [32,42]. As a lot of ATP are generated in the corneal endothelium, and lead to oxidation [43], NOX4 may be involved in corneal-endothelial function. NOX4 is known to be constitutively active and involved in the redox regulation of cell homeostasis [44]. In this study, we revealed that cryoinjury on the corneal endothelium of SD rats caused corneal opacity and reduced cell density, which was ameliorated by NOX4 inhibition. Notably, cryoinjury mimics bullous keratopathy, though the severe injury of CEnCs [45]. Moreover, NOX4 suppression protected the CEnCs against cryoinjury-induced damage, and promoted the wound-healing of the corneal endothelium after cryoinjury, which restored corneal transparency. In this study, we did not analyze the expression of NOX4 in the retina. Performing gene transfer into corneal-endothelial cells is confined to the anterior chamber, with electroporation creating the directionality of the gene, and the aqueous humor facilitating flow [46]. Thus, it is unlikely that the gene entered into the retina or affected it directly. NOX4 plays an important role in angiogenesis and fibrosis in the wound-healing process [22,47]. NOX4 triggers a pathological response in alkali injury of the cornea, and the inhibition of NOX4 reduces corneal inflammation in alkali injury [48]. NOX4 promotes epithelial-to-mesenchymal transition (EMT) and fibrosis [49,50], and the suppression of EMT promotes the regeneration of CEnCs, by inhibiting the TGF-β/ROS signaling pathway [51,52]. During the wound-healing process, TGF-β regulates cell proliferation, the transdifferentiation from fibroblast to myofibroblast, the production of the extracellular matrix, and the immune response [53,54]. TGF-β binds to transmembrane TGFβ receptor II (TGFβRII), followed by phosphorylation through the serine/threonine kinases of transmembrane TGFβ receptor I (TGFβRI) [55,56]. Activated TGFβRII phosphorylates Smad2 or Smad3, which translocate into the nucleus. Activated Smad complexes bind DNA via transcriptional factors in the nucleus [57]. TGF-β receptors can activate several independent signaling pathways, through direct interaction or phosphorylation [58]. Three of the main signaling pathways activated by TGF-β receptors are the mitogen-activated protein kinase (MAPK) pathway, the Rho-like GTPase pathway, and the phosphatidylinositol-3-kinase (PI3K) pathway [58]. TGF-β also triggers intracellular ROS release by the upregulation of NOX4 [59]. NOX4 generates ROS, and is involved in redox signaling [60]. NOX4 and redox signaling mediate the TGF-β-induced phenotypic switch via the p38/AKT signaling pathway [60]. Oxidative stress induced by NOX4 may activate HIF-1α and MMP-9 via the NF-kB signaling pathway [61], which plays an essential role in connection between oxidative stress and cellular responses [62].

We performed in vitro experiments to understand the mechanism underlying this phenomenon in vivo. Our results revealed that NOX4 regulates the cell shape, viability, and proliferation of CEnCs. Corneal-endothelial diseases are characterized by enlarged cells and a change in shape, which can lead to endothelial dysfunction, similar to the senescence that occurs when NOX4 generates ROS [63]. This change in phenotype may be due to the overexpression of the NOX4 gene, which is a member of the NADPH oxidase family [35]. The NOX4 enzyme is responsible for producing superoxide and hydrogen peroxide, which are reactive oxygen species that can cause oxidative stress [64]. Oxidative stress can cause cell damage and changes in the morphology of cells, which is likely why the transfected cells adopted a fibroblast-like phenotype [60]. ROS are involved in cell reprogramming for proliferation and intracellular signaling [65]. NOX4-derived ROS mediate transforming growth factor-beta1 (TGF-β1)-induced metabolic reprogramming during epithelial-mesenchymal transition, through the phosphatidylinositol 3-kinase (PI3K)/protein kinase B (AKT)/hypoxia-inducible factor 1 pathway [66]. Additionally, NOX4 regulates proliferation. Therefore, the excessive generation of ROS by NOX4 overexpression inhibits the proliferation of CEnCs through senescence. Senescence can occur spontaneously in cultured cells, including corneal-endothelial cells, over time [67]. Senescence is mediated by activating the extracellular signal-regulated protein kinase (ERK)1/2 signaling pathways, and the small GTPase Rho [68]. NOX4-derived-ROS induces senescence through the mitogen-activated protein kinase and NF-κB pathways [69]. Senescence is characterized by irreversible cell-cycle arrest and SA-β-gal. In this study, we found that NOX4 regulates senescence in hCEnCs via regulating oxidative stress.

In addition, our results revealed that NOX4 regulates ER stress in hCEnCs. NOX4 is mainly localized in the ER, and NOX4-derived ROS leads to ER stress [70], which is characterized by swelling and enlargement [71]. ER stress can be activated via three pathways; however, ROS-induced ER stress mainly occurs via the ATF4 pathway and the ATF6 pathway [72]. ATF4 is a stress-induced transcription factor that is upregulated during oxidative and ER stress [73]. In response to ER stress, ATF4 migrates to the nucleus, and activates genes [73,74], and ATF6 induces XBP1 mRNA, which is spliced to produce a highly active transcription factor [75]. Then, XBP1 migrates to the nucleus, and regulates target genes encoding ER molecular chaperones [76]. ER stress and senescence are closely linked processes [77]. Persistent or severe ER stress can induce cellular senescence through the activation of the UPR-including ATF4/6/XBP-1 pathways [78]. UPR can lead to the activation of the transcription factor p53, which is a key regulator of senescence [79]. The accumulation of senescent cells with dysfunctional ER contributes to tissue dysfunction [80].

In this study, we revealed that NOX4 regulates mitochondrial membrane potential and autophagy. A previous study reported that NOX4 inhibits mitochondrial bioenergetics, and NOX4 depletion decreases mitochondrial swelling, cytochrome c release, and mitochondrial DNA (mtDNA) damage [81]. Mitochondrial respiratory chain complex I, which plays an important role in energy production, is inactivated by NOX4 [82]. NOX4 induces mitochondrial dysfunction, while NOX4 depletion stabilizes the mitochondrial membrane potential, and induces changes in the mitochondrial shape [82]. The mitochondrial membranes are mildly depolarized and coupled with the respiratory synthesis of ATP [83]. NOX4 is a major source of oxidative stress in mitochondria, and produces oxidative stress [84]. Excessive oxidative stress affects mitochondrial functions, coupled with mitochondrial DNA damage, the oxidation of mitochondrial proteins, and a change in the mitochondrial membrane potential [84]. The mitochondrial membrane potential is hyperpolarized in early response to oxidative stress [85,86], which is associated with mitochondrial dysfunction [87], and then depolarized, which is a signal of bioenergetic stress [88]. The depolarization of the mitochondrial membrane leads ATP production to stop; it also leads to the release of cytochrome c, and the induction of apoptosis, although the mild depolarization of the inner mitochondrial membrane is important in an anti-aging program, via attenuating mitochondrial reactive oxygen species [83]. The upregulation of NOX4 has been reported to induce depolarization of the mitochondrial membrane [89], although hyperpolarization of the mitochondrial inner membrane has been reported to precede excessive generation of ROS [90]. Additionally, NOX4 inhibition improves mitochondrial function and survival [91]. NOX4-mediated ROS production induces apoptotic cell death via the downregulation of c-FLIP and Mcl-1 expression [24]. Moreover, NOX4 and redox signaling mediate TGF-β-induced endothelial cell apoptosis and phenotypic switch [92]. NOX4 plays an essential role in regulating autophagy via activating the ROS/PERK/eIF-2α/ATF4 pathway [93,94], and its activation in the ER promotes autophagy [95]. These results are consistent with our findings that NOX4 regulates the mitochondrial potential and autophagy of hCEnCs. In this study, we found that oxidative stress generated by NOX4 induces senescence, ER stress, and autophagy. ER stress participates in the progress of cellular senescence [96], and is involved in the tethering of the ER to the mitochondria, resulting in an increase of mitochondrial calcium intake and ROS production [57]. The p38-activated ER stress mediates cellular senescence [97]. Autophagy and senescence are induced by common stressors, including oxidative stress and DNA damage [98]. Autophagy regulates senescence, while senescent cells can alter autophagic activity [98]. Autophagy plays a role in regulating the induction and maintenance of senescence by p38α signaling [99]. Autophagy forms autophagosomes, which fuse with lysosomes to form autolysosomes [100]. Lysosomes play an important role in senescence [100]. mTOR, an essential signaling pathway for autophagy, is recruited to the lysosome, activates the lysosome, and is essential for initiating the secretion of SASP factors [100]. Although autophagy was initially thought to be a process counteracting senescence by removing damaged organelles, autophagy upregulation is required for the implementation of oncogene-induced senescence [101]. NOX4 activity leads to mitochondrial fragmentation, and increased mitochondrial-outer-membrane permeability, which in turn triggers autophagy. The NOX4-mediated activation of the mitochondrial fission protein dynamin-related protein 1 (DRP1) is required for the induction of autophagy.

## 5. Conclusions

This study revealed that NOX4 regulates the wound-healing and senescence of hCEnCs, by modulating oxidative stress, ER stress, and autophagy. Thus, the regulation of NOX4 may be a potential therapeutic strategy for regulating the homeostasis of CEnCs, and treating corneal-endothelial diseases.

## Data Availability

Data are available from the corresponding author on reasonable request.

## Supplementary Materials

The following supporting information can be downloaded at: https://www.mdpi.com/article/10.3390/antiox12061228/s1, Table S1: Primers for RT-PCR.
