# Supplementary material for: Role of NADPH Oxidase 4 in Corneal Endothelial Cells Is Mediated by Endoplasmic Reticulum Stress and Autophagy
Source: Antioxidants (Basel). 2023 Jun 7;12(6):1228. doi: 10.3390/antiox12061228 (PMC10294998; doi:10.3390/antiox12061228)
Supplement: Supplementary file 1 [file antioxidants-12-01228-s001.zip › antioxidants-2390881-supplementary.pdf]

Table S1. Primers for RT-PCR

Gene<br>(Gene accession No.)	Primer	Primer sequence (5′ to 3′)
NOX4 (NM_001143836)	Forward	GACTTTACAGGTATATCCGGAGCAA
	Reverse	TGCAGATACACTGGACAATGTAGA
β-actin (NM_001101)	Forward	AGAGCTACGCTGCCTGAC
	Reverse	AGCACTGTTGGCGTACAG
ATF6	Forward	TTGGCATTTATAATACTGAACTATGGA
	Reverse	TTTGATTTGCAGGGCTCAC
GAPDH	Forward	CTGGGCTACACTGAGCACC
	Reverse	AAGTGGTTGAGGGCAATG
